# Role of dyslipidemia in early vascular aging syndrome

**DOI:** 10.3906/sag-2008-165

**Published:** 2021-04-30

**Authors:** Alparslan KILIÇ 1, Onur BAYDAR, Deniz ELÇİK, Ziya APAYDIN, Mehmet Mustafa CAN

**Affiliations:** 1 Department of Cardiology, Koç University Hospital, İstanbul Turkey; 2 Department of Cardiology, Faculty of Medicine, Erciyes University, Kayseri Turkey; 3 Department of Cardiology, Haseki Training and Research Hospital, İstanbul Turkey

**Keywords:** Arterial stiffness, vascular aging, lipid profile

## Abstract

**Background/aim:**

Arterial stiffness, known as a predictor of early vascular aging, was defined as the main determinant of cardiovascular mortality and morbidity. However, the relationship between lipid profile and increased arterial stiffness is not clear. The aim of this study is to investigate the relationship between lipid profiles and increased arterial stiffness in patients with early vascular aging syndrome.

**Materials and methods:**

A total of 1582 participants —504 (31.8%) of were male and the mean age was 52.8 ±14.2 years— were included in the study . Patients who applied to the hospital for various reasons and who had undergone 24-h blood pressure Holter monitoring were included in this study. Patients were divided into four groups according to pulse wave velocity (PWV) quartiles (Q1 (<6.3), Q2 (6.3–7.4), Q3 (7.5–8.8), Q4 (>8.8)).

**Results:**

We found that in the highest PWV group, patients had higher systolic blood pressure (SBP), diastolic blood pressure (DBP), glucose, blood urea nitrogen (BUN), creatinine, urinary albumin excretion (UAE), uric acid(UA), total cholesterol (TC), low-density lipoprotein ( LDL-C), triglycerid (TG), and non- high-density lipoprotein (HDL-C ) levels. Additionally, diabetes mellitus (dm), age, non-HDL-C, and TG/ HDL-C levels were detected as independent risk factors of increased PWV in ordinal logistic regression analysis.

**Conclusion:**

Our study demonstrates that lipid parameters are strongly correlated with increased PWVvalue and early vascular aging. In daily clinical practice, TG\HDL-C ratio, known as atherogenic index, might be used routinely for predicted of early vascular aging and subclinical atherosclerosis.

## 1. Introduction

In recent years arterial stiffness, known as a predictor of early vascular aging syndrome(EVAS), was defined as the main determinant of cardiovascular mortality and morbidity. Arterial stifness can be measured by pulse wave velocity (PWV), which is a simple, sensitive and noninvasive method [1–4]. Arterial stiffness measurement might be associated with many fixed (age, sex, and family history) and modifiable factors (smoking, dyslipidemia, hyperglycemia, hypertension, etc.) [5,6]. It is well-known that age and hypertension are the main causes of arterial stifness [7]. Additionally, Hwang et al. [8] have showed that, regardless of age and blood pressure (BP), patients with diabetes mellitus had higher PWV, whereas patients with prediabetes had similar PWV when compared to subjects with normal fasting blood glucose. Moreover, a recent study showed that small changes in fasting glycaemia in metabolic syndrome patients free of diabetes and hypertension are associated with vascular impairment [9]. Dyslipidemia has been recognized as an independent modifiable risk factor for cardiovascular disease (CVD) and increased arterial stiffness. Previous studies indicate that dyslipidemia and arterial stiffness is associated with various mechanisms, such as the development of atherosclerotic plaques, oxidative stress, local and systemic inflammation, endothelial dysfunction, and low bioavailability of nitric oxide [10]. Dyslipidemia is typically defined as high total cholesterol (TC), low-density lipoprotein cholesterol (LDL-C), and triglyceride (TG) levels as well as low high-density lipoprotein cholesterol (HDL-C) levels [1,11,12]. Arterial stiffness is a tissue marker of subclinical atherosclerosis; therefore, the classification of lipid profile in patients with high arterial stiffness level might provide an important clinical benefit before the development of clinical atherosclerosis.

Zhan et al. showed that non-HDL-C, TG, and TC levels were associated with arterial stiffness and HDL-C level was inversely associated with arterial stiffness in Chinese population with hypertension [6]. Another prospective study reported that high triglyceride and low high-density lipoprotein cholesterol (HDL-C) levels are at the greatest risk factors for CVD in patients with essential hypertension [13]. 

 Variation of the lipid levels, such as a high plasma triglyceride level and a low HDL-C level is an early sign of underlying metabolic syndrome. Relationship of this condition with CVD, are crucial for increased arterial stiffness compared to matched age and sex group [14]. To the best of our knowledge, there are no studies with the relationship between lipid profiles and arterial stiffness in patients with EVAS in Turkish population. Therefore, the purpose of this study to investigate the characteristic features of dyslipidemia in patients with EVAS.

## 2. Materials and methods 

### 2.1. Study population 

All participants in this study were a subset of another study whose title is ‘Understanding Vascular Age: Are Clinical scoring systems useful for Early Vascular Aging Syndrome Prediction ?’ [15]. This study showed that CHADS2, CHA2DS2-VASc, and CHA2DS2VASc-HS [congestive heart failure (C), HT (H), age (A), diabetes mellitus (D), and stroke (S), vasculary disease (vasc), hyperlipidemia (H), smoke (S)] scores were positively correlated with PWV values. Our study was designed as a retrospective observational study. Patients who applied to the Haseki Training and Research Hospital, Department of Cardiology for various reasons between March 2015 and May 2018 and who had undergone 24-h blood pressure Holter monitoring were included in this study. The study protocol was approved by the local ethics committee. A total of 2796 patient files were scanned. After excluding patients with missing lipid profiles (n = 198), missing PWV data (n = 379), and patients who have taken antihyperlipidemic drugs (n = 637) the files of a total of 1582 participants were analyzed (Figure). Because the lipid profiles could be affected by these conditions, patients who take antihyperlipidemic drugs were excluded from the study (patient recipes and previously entered diagnoses were screened in an electronic environment through the hospital information management system. Patients who were previously prescribed with ICD code E78 were excluded from the study). Other exclusion criteria were defined as end-stage hepatic failure, end-stage renal impairment (dialysis patients and gfr < 30), arrhythmias (atrial fibrillation and frequent ventricular premature beats), valvular heart disease, collagen tissue diseases, malignancy, and any sleeping disorder.

**Figure:**
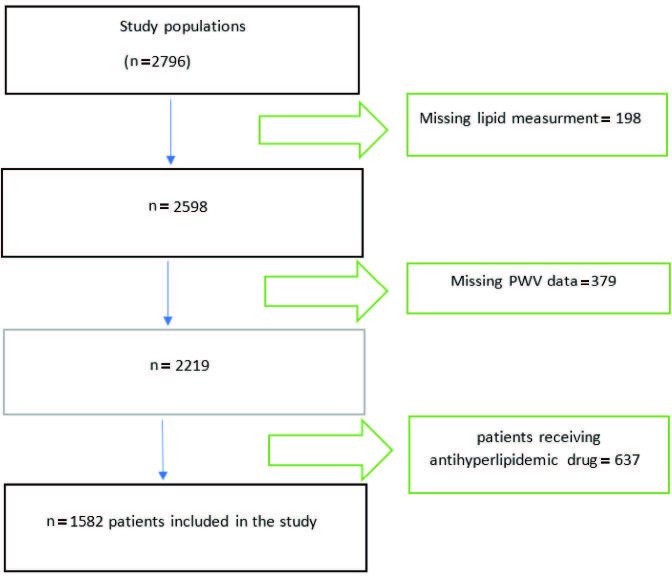
Study design

### 2.2. Clinical data collection

The demographic and clinical data of the patients in the study are composed of sex, age, body mass index (BMI), current cigarette smoking, hypertension (HT), hyperlipidemia (HL) (newly diagnosis or who did not take antihyperlipidemic drugs), coronary artery disease (CAD), heart failure (HF), family history, peripheral artery disease (PAD), stroke history, diabetes mellitus (DM), chronic renal failure, echocardiographic examination, laboratory measurements, and digital and/or nondigital hospital records. Current smoking status was defined as smoking one or more cigarettes per day for at least 1 year. According to the European Society of Cardiology guidelines, patients who have office blood pressure (BP) >140/90 mmHg or 24-h ambulatory BP >130/80 mmHg are defined as hypertensive. According to the American Diabetes Association guidelines, diabetes mellitus (DM) was defined by a previous diagnosis and/or fasting blood glucose >126 mg/dL or the use of hypoglycemic agents. HL was defined as a TC concentration >200 mg/dL, TG concentration was >150 mg/dL, LDL-C concentration >120 mg/dL, HDL-C concentration was <40mg/dL. HF was defined as a reduced left ventricular ejection fraction (<40%). PAD was defined as stenosis of at least 50% in the peripheral arterial circulation. We defined family history as the presence of heart disease or sudden cardiac death in a male first-degree relative aged < 55 years or in a female first-degree relative aged < 65 years. Stroke and transient ischemic attack (TIA) were considered as a previous stroke, which originates from carotid-vertebral system thromboembolism. Concentrations of fasting blood glucose, TG, TC, HDL-C, LDL-C, and plasma creatinine were measured by using Roche enzymatic assays (Roche Diagnostics GmbH, Mannheim, Germany) on a Roche autoanalyzer. Non-HDL-C was calculated as TC -HDL-C. Creatinine values of 1.5 mg/dL for men and 1.3 mg/dL for women were considered elevated. The patient’s laboratory information, fasting glucose levels, creatinine levels, fasting serum lipid status were noted. BMI was calculated according to the following formula weight (kg)/height (m)².

### 2.3. Assessment of arterial stiffness

In this study, office BP, ambulatory BP, PWV, and other arterial stiffness parameters were measured by using a Mobil-O-Graph Arteriograp (I.E.M. GmbH, Stolberg, Germany). Measurement of arterial stiffness and office BP were conducted in a quite environment in the morning at a stable temperature. All participants were asked to avoid alcohol, smoking, and caffeine for at least 12 h before measurement. Office BP and arterial stiffness measurements of each participant were taken after the patients had rested at least 5 min in a seated position. This measurement was made twice before and after the ambulatory Holter, and the average of 2 measurements was taken. Twenty-four hour of ambulatory BP measurements were obtained at 20 min intervals (08.00–22.00 h) during the day and at 30 min (22.00–08.00 h) intervals at night. Measurements were calculated automatically by the device and computer software. Mobil-O-Graph arteriograph, an oscillometric device that supplies simultaneous measurement of brachial BP, PWV, and augmentation index (AIx). It makes a recording of the brachial artery pressure waveform to synthesize the central pulse wave by applying a transfer function. These data are combined to estimate the aortic impedance using a special mathematical model and demographic data like age, BMI, and central pressure. In fact, there is no direct measurement of PWV. Invasive PWV is estimated via aortic characteristic impedance, which is calculated from an estimated pressure waveform and an estimated flow waveform.

These measurements included 24 h, day and night central- peripheral systolic-diastolic BP and pulse pressures, cardiac output (CO), peripheral resistance (PR), stroke volume (SV), nocturnal decreases in systolic BP and nocturnal decreases in diastolic BP, aortic stiffness, aortic augmentation pressure, AIx, PWV.

## 3. Statistical analysis

Continuous variables were described using means with standard deviations (SDs), and categorical variables were given as numbers and percentages. The participants were stratified by quartiles of PWV. Comparisons of proportions were done by chi-square test, whereas correlation analyses were performed by the Pearson rank test. The comparison of continuous variables among more than two groups were done with one-way ANOVA variance analysis. Tukey’s post hoc analysis were done. Ordinal logistic regression test was applied to determine the independent factors predicting PWV. Statistical significance was assumed at P < 0.05. Statistical analysis was performed using SPSS (USA) for MS Windows, version 22 (IBM Corp., New York, Armonk, NY, USA). 

## 4. Results

A total of 1582 participants were analyzed, 504 (31.8%) of which were male; the mean age was 52.8 ±14.2 years. The mean SBP and DBP were 126.0 + 29.4 mm Hg and 77.9 + 10.7 mm Hg, respectively. Of all participants, 23.4% were current smokers. 

We further assigned participants into subgroups using PWV quartiles (Q1 (<6.3), Q2 (6.3–7.4), Q3 (7.5–8.8), Q4 (>8.8) ). We found that, in the highest PWV group, patients had higher SBP, DBP, glucose, BUN, creatinine, urinary albumin excretion (UAE), uric acid (UA), TC, TG, LDL-C and nonHDL-C levels and higher rates of DM, stroke, HT, CAD, chronic renal failure (CRF). Also, the highest PWV was observed in older patients and the male patients (Table 1,2,3). End organ damage was statistically higher in patients with high PWV (CAD, CRF, stroke).

**Table 1:** Baseline characteristics of study participants according to PWV quartiles.

Pulse wave velocity, m/s					
Variables	Q1 (<6.3)	Q2 (6.3–7.4)	Q3 (7.5–8.8)	Q4 (>8.8)	P value
N (1582)	374	413	396	399	
Male, n (%)	86 (23%)	97 (23.7%)	95 (24.1%)	115 (29.2%)	0.1
Age, years	34.9 ± 8.1	48.7 ± 5.5	56.8 ± 5.3	69.7 ± 8.0	<0.001
BMI, kg/m2	28.3 ± 8.4	30.3 ± 5.3	30.4 ± 5.1	29.8 ± 6.2	<0.001
HT	18,7%	22%	28%	31.3%	<0.001
DM	14,7%	15,9%	31%	38.4%	<0.001
HL	13.1%	26.4%	27.9%	32.5%	<0.001
CAD	8.1%	20.3%	26.6%	45%	<0.001
CRF	10.4%	26%	20.8%	42.9%	<0.001
Previous stroke or TIA	14.3%	13.3%	28.6%	43.8%	<0.001
PAD	22.2%	16.7%	16.7%	44.4%	0.2
Current smoking	38.6%	27.4%	19.9%	14.1%	<0.001
Office SBP (mmHg)	116 ± 12.6	122 ± 13.5	131 ± 16.4	137 ± 21.1	<0.001
Office DBP (mmHg)	75 ± 11.1	80 ± 11.6	84 ± 13.2	82±14.9	<0.001
24-h SBP (mmHg)	122.6 ± 53.1	124.4 ± 13.8	127.6 ± 15.6	129.3 ± 17.9	0.007
24-h DBP (mmHg)	74.9 ± 9.7	78.6 ± 10.2	79.8 ± 11.0	78.0 ± 11.3	<0.001
24-h MAP (mmHg)	95.5 ± 10.4	101.9 ± 48.5	104.1 ± 49.5	104.0 ± 50.4	0.02
24- Heart rate (beat/min)	79.0 ± 10.4	75.9 ± 9.6	73.8 ± 9.1	73.7 ± 10.4	<0.001
24-PP (mmHg)	45.0 ± 8.7	45.7 ± 8.0	47.7 ± 9.4	51.3 ± 11.1	<0.001
Daytime SBP (mmHg)	121.4 ± 14.1	128.3 ± 51.8	129.0 ± 16.5	130.1 ± 19.2	<0.001
Daytime DBP (mmHg)	76.7 ± 10.1	80.3 ± 10.6	81.2 ± 11.6	79.3 ± 11.6	<0.001
Daytime MAP (mmHg)	97.2 ± 10.6	101.3 ± 11.7	103.3 ± 12.6	104.8 ± 49.6	0.001
Daytime Heart rate (beat/min)	81.5 ± 11.1	78.5 ± 10.3	76.1 ± 9.5	75.7 ± 10.8	<0.001
Daytime PP(mmHg)	44.9 ± 9.0	45.7 ± 8.2	47.8 ± 9.5	53.2 ± 43.0	<0.001
Nighttime SBP (mmHg)	114.3 ± 14.1	119.2 ± 14.7	125.2 ± 62.8	125.4 ± 19.4	<0.001
Nighttime DBP (mmHg)	69.0 ± 10.1	73.2 ± 10.5	74.4 ± 11.8	73.5 ± 11.7	<0.001
Nighttime MAP (mmHg)	89.7 ± 11.2	94.1 ± 12.4	96.2v13.9	103.2 ± 85.4	<0.001
Nighttime Heart rate (beat/min)	69.6v9.9	67.8 ± 8.9	66.4 ± 8.8	67.2 ± 9.7	<0.001
Nighttime PP (mmHg)	45.2 ± 9.0	46.0 ± 8.6	47.7 ± 10.0	51.9 ± 12.1	<0.001
The rate of systolic fall in nighttime (%)	5.9 ± 0.3	5.3 ± 0.3	5.5 ± 0.4	3.5 ± 0.3	<0.001
The rate of diastolic fall in nighttime (%)	8.9 ±.1.7	8.8 ± 1.7	8.7 ± 1.8	8.4 ±.1.9	0.008
Central systolic blood pressure (mmHg)	107.5 ± 11.7	114.4 ± 12.9	120.6 ± 16.3	125.1 ± 19.6	<0.001
Central diastolic blood pressure (mmHg)	76.7 ± 11.2	82.0 ± 11.8	85.1 ± 13.7	84.0 ± 14.8	<0.001
Central pulse pressure (mmHg)	30.8 ± 7.6	32.3 ± 7.7	35.4 ± 9.2	41.1 ± 11.4	<0.001
Cardiac output (l/min)	4.8 ± 0.7	4.9 ± 0.7	5.2 ± 0.8	5.2 ± 0.9	<0.001
Cardiac index (l/min*1/m²)	2.4 ± 0.5	2.4 ± 0.4	2.6 ± 0.5	2.6 ± 0.5	<0.001
AIx (%)	23.8 ± 12.2	25.0 ± 13.1	23.6 ± 13.4	28.7 ± 14.2	<0.001
cAIx (%) n (%)	25%	20.7%	24.7%	29.6%	0.08

**Table 2:** Baseline Laboratory results of study participants according to PWV quartiles.

Pulse Wave Velocity, m/s					
Variables	Q1 (<6.3)	Q2 (6.3-7.4)	Q3 (7.5-8.8)	Q4 (>8.8)	P Value
Glucose (mg/dl)	103.6±42.0	107.4±31.4	116.5±55.8	122.2±55.2	<0.001
BUN (mg/dl)	26.0±8.7	29.4±9.1	31.8±11.0	36.2±14.6	<0.001
Creatinine (mg/dl)	0.83±0.2	0.88±0.37	0.91±0.32	0.96±0.34	<0.001
Creatinine clearance (ml/min)	98.9±19.8	86.2±16.9	80.5±16.4	72.4±19.1	<0.001
UAE n (%)	21.3%	18.1%	19.4%	41.2%	<0.001
Uric acid (mg/dl)	4.8±1.4	5.1±2.0	5.1±1.3	5.5±1.6	<0.001
AST	22.3±15.7	22.7±10.1	22.8±12.0	22.3±10.2	0.6
ALT	22.4±16.1	23.2±15.2	23.3±15.2	20.1±14.2	0.1
Total bilirubin	0.55±0.29	0.56±0.29	0.60±0.31	0.58±0.31	0.1
HBG (mg/dl)	13.8±1.5	13.7±1.6	13.8±1.5	13.5±1.5	0.2
HTC (%)	41.9±4.5	41.6±4.6	41.9±4.4	41.3±4.4	0.5
WBC (K/ul)	8.1±2.3	7.9±2.2	7.7±2.2	7.8±2.2	0.01
Neutrophils (mm3 )	5.0±2.0	4.9±1.8	4.6±1.7	4.8±1.8	<0.001
Lymphocytes (mm3	2.3±0.7	2.2±0.6	2.2±0.7	2.1±0.7	0.03
PLATELET (×103 /L)	271.9±70.3	276.7±65.0	266.9±66.9	250.1±65.5	<0.001
MPV (fL)	9.1±1.3	9.2±1.3	9.0±1.2	9.2±1.3	0.07

**Table 3:** Baseline lipid parameters of study participants according to PWV quartiles.

Pulse wave velocity (PWV), m/s					
Variables	Q1 (<6.3)	Q2 (6.3–7.4)	Q3 (7.5–8.8)	Q4 (>8.8)	P value
TC (mg/dL)	188.2 ± 41.3	203.2 ± 40.1	205.5 ± 46.7	206.7 ± 43.5	<0.001
Triglyceride (mg/dL)	153.3 ± 112.9	168.6 ± 99.1	170.3 ± 99.5	179.1 ± 127.7	0.013
HDL-C (mg/dL)	47.9 ± 12.0	47.8 ± 11.7	48.7 ± 12.5	48.8 ± 12.4	0.51
LDL-C (mg/dL)	111.1 ± 35.4	122.3 ± 33.6	123.0 ± 37.7	125.1 ± 36.8	<0.001
NonHDL-C (mg/dl)	141.4 ± 40.9	156.1 ± 36.8	157.3V42.3	159.3 ± 47.7	<0.001
TC/HDL-C	4.1 ± 1.2	4.4 ± 1.2	4.4 ± 1.2	4.4 ± 1.2	0.001
TG/HDL-C	3.6 ± 0.18	3.9 ± 0.17	3.8 ± 0.13	4.1 ± 0.22	0.02
LDL-C/HDL-C	2.3 ± 0.9	2.6 ± 0.9	2.6 ± 0.9	2.6 ± 1.0	0.001
Non–HDL-C/HDL-C	3.1 ± 1.2	3.4 ± 1.1	3.4 ± 1.2	3.4 ± 1.1	0.001

PWV levels were significantly correlated with age (r: 0.931, P < 0.001), BMI (r: 0.166, P < 0.001), TC (r: 0.139, P < 0.001), LDL-C(r: 0.119, P < 0.001), TG( r: 0.099, P < 0.001), Non- HDL-C (r: 0.140, P < 0.001) and TG\ HDL-C ratio (r: 0.083, P: 0.001).

In 24 h of ambulatory BP measurement, central systolic BP, central diastolic BP, central pulse pressure, cardiac output, cardiac index, and AIx were statistically significant among the groups. In addition, a statistically significant difference was found in the PWV-high group in all 24-h ambulatory parameters (Table 1).

Metabolic syndrome and atherosclerosis factor LDL/HDL and total cholesterol/HDL were significantly higher in patients with high PWV (P = 0.001).

Additionally, DM (OR: 0.76(0.25–1.02), P: 0.007), age (OR: 1.07 (0.39-1.08), P < 0.001), non-HDL-C (OR: 0.99 (0.36–1.0), P: 0,002 )and TG/ HDL-C levels (OR: 0.97 (0.34–1.05), P: 0.043) were detected as independent risk factors of increased PWV in ordinal logistic regression analysis (Table 4).

**Table 4:** Independent risk factors of PWV in ordinal logistic regression analysis.

Variables	OR (95% C.I)	P
Age	1.07 (0.39–1.08)	<0.001
Sex	0.83(0.027–1.08)	0.16
BMI	1.0 (0.36–1.02)	0.84
DM	0.76(0.25–1.02)	0.007
Current Smoking	0.93 (0.31–1.24)	0.64
TG	1.0(0.36–1.0)	0.24
Non-HDL C	0.99 (0.36–1.0)	0.002
TG/HDL-C	0.97 (0.34–1.05)	0.043

## 5. Discussion

The main results of our study showed that PWV levels were significantly correlated with age, BMI, TC, LDL-C, TG, non-HDL-C and TG\ HDL-C ratio (2). Additionally, factors like DM, age, non-HDL-C, and TG/ HDL-C levels were shown to be independent risk factors of increased PWV in ordinal logistic regression analysis.

Non-HDL-C, which is known as the main atherogenic cholesterol, included very low-density lipoprotein (VLDL) cholesterol, apolipoprotein B, lipoprotein a, and intermediate-density lipoprotein cholesterol [16]. These forms of cholesterol are known to be the main contributors for CAD development [17]. Cholesterol-rich residue inside of the non-HDL-C fraction can pass the endothelial wall and provoke foam cell formation [16]. Poledne et al. were showed that non-HDL-C level is associated with the number and phenotype proportion of macrophages in visceral adipose tissue [18]. Zhan et al. demonstrated that non-HDL-C was associated with arterial stiffness in a Chinese population with hypertension [6]. In addition, Ito et al. showed that one standard deviation increase in non-HDL-C was linked with 37% increased risk of incident CAD [16]. Similar to the literature, we found that PWV levels were significantly correlated with non-HDL-C levels. Also, non-HDL-C is an independent risk factor of increased PWV in ordinal logistic regression analysis.

Triglycerides, which are transferred by TG-rich lipoproteins, might create reactive oxygen radicals, which leads to development of insulin resistance and more importantly, increased TG levels could start proatherogenic pathways [19]. Recently, some studies have shown that increased TG level is an additional risk for cardiovascular disease [20]. In this study, we detected that increased TG levels were linked with an elevated level of PWV. There may be a few reasons for these conditions. TG level is linked with smaller LDL particles, which are more atherogenic; therefore, it easily passed into the vessel endothelium and provoke an inflammatory reaction [21]. Second, TG metabolism is associated with apolipoprotein (Apo) C-III level. Increased ApoC-III levels can cause decreased TG clearance and elevated LDL particle formation. The combination of these factors, creates abnormal arterial smooth muscle and abnormal endothelial vasodilatory response [21,22]. 

On the other hand, HDL-C, which is the main antiatherogenic lipoprotein, provokes excess cellular cholesterol to join the bloodstream and accelerates reverse cholesterol transport to the liver [23]. Other effects of HDL-C on the metabolism protect against atherosclerosis by restricting lipoprotein oxidation. The antioxidant features of HDL depend on the serum paraoxonase and esterase activity [24]. The general opinion in literature on the effects of HDL-C are that lower levels are linked with an increased risk of CAD [25]. Some studies that are intended to increase HDL-C levels to decrease the risk of CAD were not successful and this debate continues [26]. In our study, we found that HDL-C is not an independent risk factor of increased PWV.

In the light of all of these mechanisms, TG-HDL-C ratio, which demonstrates the ratio between the atherogenic and preventive lipoproteins, might be an effective predictive value for detecting the early vascular aging and subclinical atherosclerosis. Furthermore, studies have demonstrated that TG-HDL-C ratio is better than classical lipid or lipid ratios in predicting insulin resistance [27]. Kimm et al. showed that TG-HDL-C proportions are significantly linked with insulin resistance in patients without metabolic syndrome in Korean populations [27]. Insulin resistance is part of CVD pathogenesis and also insulin resistance was independently associated with increased PWV [28]. Wen et al. showed that lipid ratios, especially TG-HDL-C ratio, are superior to conventional lipid parameters for predicting arterial stiffness in young Korean men [24]. The underlying mechanisms of the relationship between insulin resistance and dyslipidemia is known as follows: First, insulin resistance causes an increased production and secretion of TG-rich LDL. Second, it reduces HDL-C levels [29]. Some researchers suggest that the TG/HDL-C ratio, which is known to predict insulin resistance, provides an indicator of arterial stiffness associated with insulin resistance [30]. McLaughlin et al. found that TG/HDL-C proportions have the predictive power to detect insulin-resistant overweight individuals with normal glucose tolerance [30]. Similar to the literature in this study, we found that increased TG/ HDL-C levels were positively correlated with increased PWV levels and independent risk factor of increased PWV. 

Our study has several limitations as follows: (1) it is a cross-sectional retrospective study; (2) the deficiency of follow-up data; (3) carotid-femoral pulse wave velocity (cfPWV) is the gold standard method for assessing arterial stiffness. The European Society of Cardiology/European Society of Hypertension has endorsed cfPWV for assessing hypertension mediated organ damage. In our study, we used an automatic oscillometric upper arm cuff BP monitor for PWV measurement. 

## 6. Conclusion 

Our study demonstrates that lipid parameters are strongly correlated with increased PWV and early vascular aging. In daily clinical practice, TG\ HDL-C ratio might be used routinely for prediction of early vascular aging and subclinical atherosclerosis. Prospective studies with long-term follow-up are obligatory to obtain more data about the relationship between PWV and TG\HDL-C ratio.

## Informed consent

Haseki Training and Research Hospital review board and approval code is 20.04.2020/ 106.
